# Molecular characterization and genetic diversity of hepatitis B virus among health care workers in the Bamenda Health District, Northwest Region, Cameroon

**DOI:** 10.1016/j.ijregi.2026.101012

**Published:** 2026-09-21

**Authors:** Etheline W. Akazong, Richard Njouom, Marie Amogou, Lawrence Ayong, Christopher Tume, Jules-Roger Kuiate

**Affiliations:** 1 University of Dschang, Dschang, Cameroon; 2 Centre Pasteur in Yaoundé, Yaoundé, Cameroon; 3 University of Bamenda, Bambili, Cameroon

**Keywords:** HBV, HBV genotype E, HBV genotype A, Health care workers, Occupational exposure

## Abstract

**Objectives:**

Hepatitis B virus (HBV) infection is endemic in Africa, with an estimated 82 million people living with chronic HBV infection. The outcome of exposure to HBV is a complex interplay between host immunity and viral factors, including HBV genotypes. Despite the high burden of HBV infection, data on HBV genotypes circulating in many African settings, including Cameroon, remain limited. This study investigated the genetic diversity of HBV among health care workers in the Bamenda Health District, Cameroon.

**Methods:**

In this cross-sectional hospital-based study, HBV DNA was extracted from plasma samples from consenting health care workers who tested positive for hepatitis B surface antigen. The partial pre-S2/S region was amplified and sequenced, and major HBV genotypes were assigned by phylogenetic analysis.

**Results:**

The S-gene region was successfully amplified and sequenced from 11 participants. Phylogenetic analysis revealed that five sequences clustered with genotype E and four with genotype A references, whereas two divergent sequences remained unresolved. No gross length polymorphism was observed among the amplified products.

**Conclusion:**

This study highlights HBV genetic diversity among HCWs in Cameroon, with genotype A– and genotype E–associated sequences represented among the eligible samples. The coexistence of these genotypes may reflect patterns of HBV exposure and transmission and provides molecular evidence on HBV genotypes circulating in this population in Cameroon.

## Highlights

• A total of 11 eligible hepatitis B virus sequences from health care workers were analyzed.
• Five sequences clustered with genotype E and four with genotype A references.
• Two divergent sequences remained unresolved in the phylogenetic analysis.
• Partial pre-S2/S sequencing supported major genotype-level assignment.
• Data support the need for hepatitis B virus vaccination and surveillance among health care workers.

## Introduction

Hepatitis B virus (HBV) is a partially double-stranded deoxyribonucleic acid (DNA) enveloped virus of the Hepadnaviridae family. HBV infects the liver, leading to hepatocellular necrosis and inflammation, and can cause either an acute self-limiting infection or a chronic persistent disease [1,2]. Chronic HBV infection can progress to serious complications such as chronic hepatitis, cirrhosis, and hepatocellular carcinoma. Worldwide, HBV remains a major public health concern, with an estimated 240 million people chronically infected and approximately 1.1 million deaths reported in 2024 due to HBV-related complications, including cirrhosis and liver cancer [2].

Despite the availability of a safe and effective vaccine, HBV continues to spread through exposure to infected blood and body fluids [3,4]. Health care workers (HCWs), whose job is to care for the sick and injured, are at an increased risk of infection due to occupational exposure, particularly in regions with high HBV endemicity. The risk of infection among HCWs is influenced by the prevalence of HBV in the general population and the frequency of percutaneous and mucosal exposures encountered during their professional activities [4].

The HBV genome, a relaxed circular DNA of approximately 3.2 kb, shows high genetic variability, with a nucleotide substitution rate estimated between 1.4 × 10⁻⁵ and 3.2 × 10⁻⁵ substitutions per site per year [5,6]. Based on an 8% divergence across the full genome or a 4% divergence in the S-gene, HBV has been classified into ten genotypes (A-J) [7,8]. These genotypes are thought to have distinct geographical and ethnic distributions and are associated with differences in clinical outcomes, disease progression, and responses to antiviral therapy [9].

Genotype A is the most widely distributed worldwide, predominant in Europe, North America, Africa, and India [10,11], whereas genotype E is mainly restricted to sub-Saharan Africa [12]. Cameroon, located in this region, is classified as hyperendemic for HBV, with an estimated national hepatitis B surface antigen (HBsAg) prevalence of 11.2% [13]. Previous research in the Northwest Region (NWR) of Cameroon reported an HBV prevalence of approximately 10.6% among HCWs, with approximately 23.8% of those infected testing positive for hepatitis B e-antigen (an infectivity marker) [14]. However, limited information exists on the HBV genotypes circulating among HCWs in this region.

This study aimed to determine the HBV genotype circulating among HCWs in the Bamenda Health District, NWR of Cameroon, using the HBV pre-S2/S region. Knowledge of circulating HBV strains and their genetic variability in this high-risk occupational group is crucial for effective disease monitoring, developing prevention strategies, and improving therapeutic outcomes.

## Methods

### Study design

This cross-sectional, hospital-based study was conducted among HCWs in the Bamenda Health District, located in the NWR of Cameroon.

### Study population

The study population (Table 1) included a diverse group of HCWs composed of 17 medical doctors, 224 nurses and midwives, 23 dentists and pharmacists, 90 laboratory technicians, and 44 sanitary workers (including cleaners, carriers, and launderers). Biomedical students accounted for 37.4% (n = 149) of participants [15].

**Table 1:** Baseline characteristics of the studied population.

Variables		Frequency (398)	Percentage
**Sex**	Male	126	31.7
	Female	272	68.3
**Age intervals**	16-25	167	42.0
	26-35	157	39.4
	36-45	50	12.6
	46-65	24	6.0
**Duration in the occupation**	≤1 year	72	18.1
	2-4 years	218	54.8
	5-9 years	55	13.8
	≥10 years	53	13.3
**HCW category**	Nurse	224	56.3
	Lab technician	90	22.6
	Medical doctor	17	4.3
	Dentist/Pharmacist	23	5.8
	Sanitary worker	44	11.1
**Role in the hospital**	Biomedical student	149	37.4
	Biomedical personnel	249	62.6
**Residence**	Urban	335	84.2
	Semi-urban	18	4.5
	Rural	45	11.3

Abbreviation: HCW, health care worker.

Data are presented as number (n) and percentage (%).

### Sample collection

Biological samples were collected over a 6-month period from April to September 2017. All HCWs working in the selected health facilities were informed about the study objectives and procedures before their participation. Approximately 3 mL of venous blood was collected into a plain (red-top) tube and another 3 mL into an ethylenediaminetetraacetic acid (purple-top) tube from each participant who provided written informed consent. A standardized questionnaire, designed by the research team, was administered to collect sociodemographic data and professional details. Each participant was assigned a unique identification code to securely link the questionnaire data with laboratory results.

### HBV serology

HBsAg was detected in serum of participants using the Monolisa HBsAg ULTRA ELISA kit (Bio-Rad Laboratories, Marnes-la-Coquette, France), which has a reported sensitivity and specificity above 99%. The enzyme-linked immunosorbent assay procedure was performed according to the manufacturer’s instructions, with optical density values interpreted following established kit cutoffs.

### Molecular analysis

Molecular characterization was performed on all HBsAg-positive samples to determine circulating HBV genotypes among HCWs. Viral DNA was extracted from 200 µL of plasma using the QIAamp DNA Mini Kit (Qiagen, Valencia, CA, USA), following the manufacturer’s protocol for purification of viral nucleic acids [16].

To amplify the HBV pre-S2/S-gene region, a semi-nested polymerase chain reaction (PCR) approach was used. In the first-round PCR, primers P2f (CCT GCT GGT GGC TCC AGT TC; positions 56-75) and 979 (ATT GGA AAG TAT GTC AAA GAA TTG TGG GTC TTT TG; positions 977-1011) were used. The semi-nested PCR was conducted with P2f as the forward primer and Mc2r (TGG AAG TTG GGG ATC ATT GCC; positions 891-911) as the reverse primer. Based on these primer coordinates, the expected size of the semi-nested PCR amplicon was approximately 856 bp.

The thermocycling conditions for both PCR reactions included an initial denaturation at 94°C for 5 minutes, followed by 40 cycles of 94°C for 20 s, 63°C for 20 s, and 72°C for 1 minute, with a final extension at 72°C for 5 minutes. PCR products were resolved on a 1.5% agarose gel to confirm amplification, and successful amplicons were purified and sent for Sanger sequencing at Genewiz (France).

### DNA sequence analysis and genotyping

Raw sequence data were edited and analyzed using BioEdit (Tom Hall, Raleigh, NC, USA) and Chromas Lite software (Technelysium Pty Ltd, South Brisbane, QLD, Australia). Sequence identities were confirmed through Basic Local Alignment Search Tool (BLAST) nucleotide searches against reference sequences in the National Center for Biotechnology Information GenBank database [17,18].

For genotyping, phylogenetic analysis was performed to determine the major HBV genotypes represented among the study sequences. The sequences deposited in GenBank were 792 nucleotides in length. The analysis included 11 study sequences and 13 reference sequences, giving a total of 24 taxa. Multiple sequence alignment and evolutionary analyses were conducted using MEGA12 (MEGA Development Team, Temple University, Philadelphia, PA, USA) [19]. Phylogenetic relationships were inferred using the Neighbor–Joining method, with evolutionary distances calculated using the Kimura two-parameter model. Branch support was assessed using 1000 bootstrap replicates. All codon positions (first, second, and third) and non-coding positions were included, and ambiguous positions were removed for each sequence pair using pairwise deletion. The final alignment comprised 702 nucleotide positions. Genotypes were assigned based on the phylogenetic clustering of the study sequences with established HBV genotype reference sequences. The analysis focused on major genotype assignment; subgenotype and recombinant classification were not undertaken because they require greater phylogenetic resolution than that provided by the pre-S2/S-gene region analyzed.

### Statistical analysis

Statistical analyses were conducted using IBM SPSS Statistics version 22.0 (IBM Corp, Armonk, NY, USA). Continuous variables were summarized using medians and interquartile ranges, whereas categorical variables were summarized as frequencies and percentages. Baseline demographic, occupational, and serological characteristics of participants with HBV sequences were examined descriptively according to phylogenetic genotype assignment. Given the small number of genotype-linked sequences, no formal inferential statistical analysis of genotype-associated characteristics was performed.

## Results

### Characteristics of participants with HBV sequences

A total of 398 HCWs were recruited from health facilities across the Bamenda Health District, as previously reported [14]. Among these, 42 (10.6%) were HBsAg-positive. The pre-S2/S-gene region was successfully amplified and sequenced from 11 participants who were included in the phylogenetic analysis.

Among the 11 participants included in the phylogenetic analysis, eight (72.7%) were female and three (27.3%) were male. Six (54.5%) were aged 26-35 years, and nine (81.8%) resided in urban areas. Nurses were the most represented professional group (45.5%, n = 5), followed by laboratory technicians (36.4%, n = 4), whereas one (9.1%) dentist and one (9.1%) medical doctor were represented. Only two participants reported previous HBV vaccination, of whom one had completed the vaccination schedule.

After phylogenetic analysis, four sequences clustered with genotype A reference sequences and five with genotype E reference sequences, whereas two divergent sequences remained unresolved. Genotype A–associated sequences were identified among female participants, whereas genotype E–associated sequences occurred among female and male participants and across different professional categories. No clear descriptive relationship was observed between genotype assignment and previous HBV testing status (Table 2).

**Table 2:** Summary of participants whose S-genes were sequenced.

SN	Age	Sex	Res	Occ	Exp. (yrs)	Vax	Full vax	Tested	Hos	Genotype
52	27	1	Rural	Nurse	3	No	No	Never	Gvt	Unresolved
213	28	0	Urban	Dent	3	No	No	Never	Pri	**E**
274	50	1	Urban	Nurse	30	Yes	Yes	≥1	Gvt	**A**
303	28	1	Urban	Nurse	4	No	No	≥1	Miss	**E**
365	21	1	Urban	LT	3	No	No	Never	Miss	**A**
375	25	0	Urban	MD	1	No	No	≥1	Miss	**E**
434	28	1	Urban	Nurse	7	No	No	≥1	Pri	**A**
443	32	1	Rural	Nurse	4	No	No	≥1	Miss	**E**
464	21	0	Urban	LT	4	No	No	≥1	Gvt	**E**
513	32	1	Urban	LT	4	Yes	Part	≥1	Gvt	Unresolved
551	28	1	Urban	LT	3	No	No	≥1	Pri	**A**

Exp. (yrs), duration of work (years); Full vax, complete vaccination; Genotype, phylogenetic genotype assignment; Gvt, government; Hos, hospital type; LT, laboratory technician; MD, medical doctor; Miss, missing; Occ, professional category: Dent, dentist; Part, partial; Pri, private; Res, residence; SN, serial number; Sex: 0, male; 1, female; Tested, previously tested for hepatitis B virus (HBV); Vax, HBV vaccination.

### HBV molecular characterization

All 42 HBsAg-positive HCW plasma samples were subjected to molecular analysis. The pre-S2/S-gene region was successfully amplified using semi-nested PCR amplification and sequenced from 11 (26.2%) participants. These 11 sequences were included in the phylogenetic analysis. Although amplification was achieved for a subset of samples, the precise reason why the remaining HBsAg-positive samples did not yield usable sequences (either because of unsuccessful amplification or sequencing) could not be determined because the HBV viral load was not quantified and sample quality was not systematically evaluated. Low HBV DNA concentrations and technical factors, including sequence variation at primer-binding sites, may have contributed to the amplification and sequencing success rate. No gross length polymorphism was observed among the successfully amplified products.

### Genotype distribution and phylogenetic analysis

Phylogenetic analysis of the 11 study sequences identified two major genotype groups. Five sequences clustered with genotype E reference sequences and four with genotype A reference sequences, whereas two sequences (MK978825.1 [52_P2F_13] and MK978834.1 [513_P2F_12]) showed divergent phylogenetic placement and remained unresolved (Figure 1).

**Figure 1:**
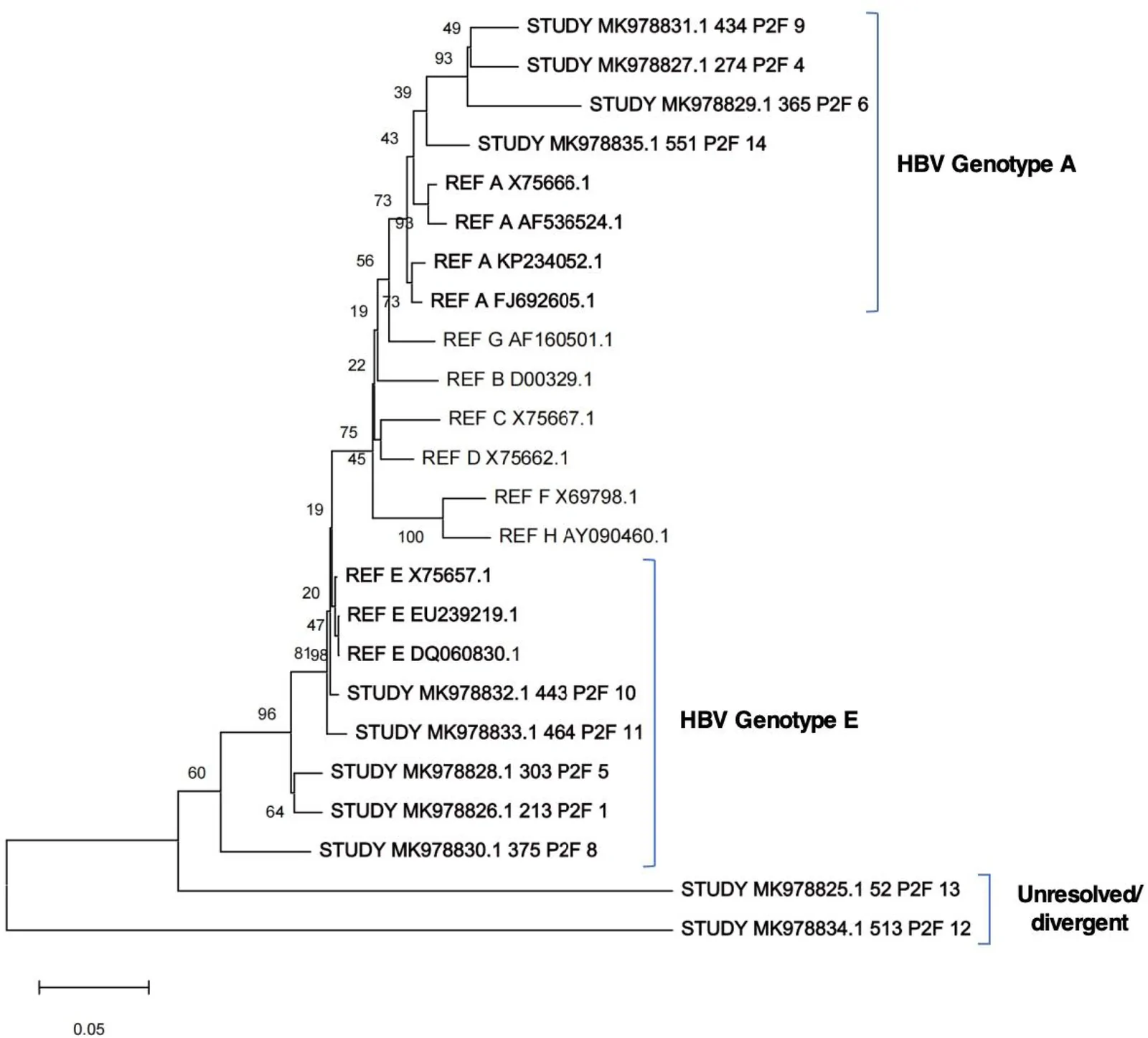
Phylogenetic analysis of HBV sequences from healthcare workers in the Bamenda Health District. The evolutionary history was inferred using the Neighbor–Joining method, with evolutionary distances calculated using the Kimura 2-parameter model. Branch support values, based on 1000 bootstrap replicates, are shown at the internal nodes. The analysis included 11 study sequences and 13 HBV genotype reference sequences (24 taxa), with 702 nucleotide positions in the final dataset. Ambiguous positions were removed using pairwise deletion. Analyses were conducted in MEGA12. HBV, hepatitis B virus.

The Neighbor–Joining phylogenetic tree showed varying levels of bootstrap support across the observed clusters. Bootstrap values are shown at the corresponding internal nodes of the phylogenetic tree and varied across branches, indicating differing levels of support for the observed clustering patterns. Overall, the phylogenetic analysis supported the presence of genotype A– and genotype E–associated HBV sequences among the HCWs studied.

The evolutionary history was inferred using the Neighbor–Joining method, with evolutionary distances calculated using the Kimura two-parameter model. Branch support values, based on 1000 bootstrap replicates, are shown at the internal nodes. The analysis included 11 study sequences and 13 HBV genotype reference sequences (24 taxa), with 702 nucleotide positions in the final data set. Ambiguous positions were removed using pairwise deletion. The analyses were conducted in MEGA12.

## Discussion

HBV infection remains a leading global cause of morbidity and mortality. The clinical outcome of HBV infection varies widely among individuals and can be influenced by viral genetics, host immune responses, environmental exposures, and other factors [20,21]. HBV can cause an acute self-limiting illness or progress to chronic infection, which may result in severe complications [22]. Worldwide, HBV accounts for more than one million deaths annually [2], showing the urgent need for improved prevention and control strategies, especially in high-burden regions such as Cameroon [13,14].

HBV transmission occurs primarily through percutaneous and mucosal exposure to infectious body fluids. Consequently, HCWs remain a high-risk group, particularly in endemic regions where vaccination coverage and post-exposure protocols may be suboptimal [23]. A previously reported study of this population found a 10.6% prevalence of current HBV infection and 47.3% total HBV exposure among HCWs [14]. Comparable studies in Cameroon reported a prevalence of 11% among HCWs in Yaoundé [24] and an alarming 18% in the Extreme North Region [25]. The prevalence of HBV infection among HCWs in this study was similar to the background prevalence of 11.5% reported in the general Cameroonian population [13], reinforcing the notion that occupational exposure reflects community-level risk. The persistence of high HBV prevalence among HCWs in Cameroon, despite the long-standing availability of vaccination, highlights the need for improved implementation of occupational vaccination programs and post-exposure prophylaxis.

### Molecular diversity and HBV genotype

HBV exhibits remarkable genetic variability and is classified into at least 10 genotypes (A-J).HBV exhibits remarkable genetic variability and is currently classified into at least 10 genotypes (A–J). Genotypes are generally differentiated by approximately 7.5–8% nucleotide divergence across the complete genome. Within genotypes, subgenotypes are commonly distinguished by 4% to less than 7.5% divergence, together with phylogenetic support [26], [27], [28]. HBV genotypes show distinct geographical distributions and have been associated with differences in transmission patterns, disease progression, response to antiviral therapy, and clinical outcome measures such as cirrhosis or hepatocellular carcinoma [20,21]. Characterizing circulating HBV genotypes, therefore, contributes to an understanding of the molecular epidemiology of HBV within affected populations. This knowledge of HBV genotype distribution is essential for disease modeling, clinical decision-making, and health resource allocation.

In the present study, phylogenetic analysis of the partial S-gene region (pre-S2/S-gene region) identified genotype A– and genotype E–associated sequences. No gross length polymorphism was observed among the amplified products; however, amplicon size alone does not measure nucleotide-level genetic diversity. Moreover, the pre-S2/S-gene region provides limited resolution for distinguishing subgenotypes and detecting recombinant viruses, so the analysis was restricted to major genotype-level interpretation.

Phylogenetic analysis of the 11 study sequences explicitly clustered five sequences with genotype E references and four with genotype A references, whereas two divergent sequences remained unresolved. The detection of genotype A– and genotype E–associated sequences is consistent with reports from West and Central Africa [20], [28], [29], [30]. Previous studies have specifically documented genotype E and genotype A, including subgenotype A3, in Cameroon [28], [29], [30]. These findings add to the evidence of HBV genetic diversity in Cameroon, while the co-circulation of these genotypes may reflect diverse patterns of viral exposure and transmission.

Descriptive examination of the participants with genotype-linked sequences showed that genotype A–associated sequences occurred among female participants, whereas genotype E–associated sequences were observed among female and male participants and across different professional categories. No clear descriptive relationship was observed between genotype assignment and previous HBV testing status. Of particular relevance to this occupational population, only two of the 11 participants reported previous HBV vaccination, and only one reported having completed the vaccination schedule. Although these findings are based on a small molecular sample and should not be generalized to the wider HCW population, the low vaccination uptake observed among these participants reinforces the importance of complete HBV vaccination among health care personnel.

### Strengths and limitations

This study provides molecular evidence of the circulation of genotype A– and genotype E–associated HBV sequences among HCWs in the Bamenda Health District and contributes to the molecular epidemiologic data available for this occupational population. The use of established genotype reference sequences and bootstrap-supported phylogenetic analysis strengthened major genotype assignment. The relatively small number of successfully sequenced samples limited genotype-stratified analyses, whereas analysis of a partial S-gene region (pre-S2/S-gene region) did not provide sufficient resolution for definitive subgenotype or recombinant classification. In addition, HBV viral load was not quantified, and sample quality was not systematically evaluated, limiting the assessment of factors contributing to unsuccessful amplification and sequencing. Future studies incorporating larger sample sizes and longer genomic regions or whole genome sequencing would enable a more detailed characterization of HBV genetic diversity and transmission patterns.

## Conclusion

The present study investigated the molecular diversity of HBV among HCWs in the Bamenda Health District, NWR, Cameroon. Phylogenetic analysis of 11 HCW-derived sequences identified genotype E– and genotype A–associated HBV sequences, with five sequences clustering with genotype E references and four with genotype A references, whereas two divergent sequences remained unresolved. The detection of both genotypes demonstrates the genetic diversity of HBV among HCWs in this population and contributes to the molecular epidemiologic evidence on circulating HBV genotypes in Cameroon. These findings support continued HBV surveillance, vaccination, and strengthened infection prevention measures among HCWs.

## Funding

This research received no specific grant from any funding agency in the public, commercial, or not-for-profit sectors. The authors wish to acknowledge Dr. A.D. Dinga Wonanke (https://orcid.org/0000-0002-9066-2715) for privately funding this study.

## Ethical approval

The study protocol conformed to the ethical guidelines of the 1975 Declaration of Helsinki. Ethical approval was obtained from the National Ethics Committee of Cameroon (N°2017/02/871/CE/CNERSH/SP). Administrative clearance to carry out this study in the NWR was obtained from the Regional Delegation of Public Health. Authorizations to access different hospitals were obtained from the directors or the persons in charge of the hospitals. Authorization to access health centers was obtained from the District Medical Officer and the chiefs of the health facilities in this region. Written informed consent was obtained from each participant before any study-related procedures were carried out.

## Data availability

Data for serological analysis: https://doi.org/10.6084/m9.figshare.13503231.v1. GenBank accession numbers of the eligible study sequences included in the revised phylogenetic analysis: MK978825.1, MK978826.1, MK978827.1, MK978828.1, MK978829.1, MK978830.1, MK978831.1, MK978832.1, MK978833.1, MK978834.1, MK978835.1.

## Declaration of generative AI and AI-assisted technologies in the manuscript preparation process

During the revision of this work, the authors used ChatGPT (OpenAI) to improve the language, clarity, and organization of the manuscript; check the consistency of terminology and references; and assist in structuring responses to reviewer comments. The authors reviewed, verified, and edited all AI-assisted output and take full responsibility for the content of the published article.

## CRediT authorship contribution statement

**Etheline W. Akazong:** Conceptualization, Data curation, Formal analysis, Funding acquisition, Investigation, Methodology, Project administration, Writing – original draft, Writing – review & editing. **Richard Njouom:** Methodology, Supervision, Writing – review & editing, Validation, Writing – original draft. **Marie Amogou:** Methodology, Writing – original draft, Writing – review & editing. **Lawrence Ayong:** Methodology, Writing – original draft, Writing – review & editing. **Christopher Tume:** Conceptualization, Methodology, Supervision, Writing – original draft, Writing – review & editing. **Jules-Roger Kuiate:** Conceptualization, Supervision, Writing – original draft, Writing – review & editing.

## Declaration of competing interest

The authors have no competing interests to declare.
